# Medical and Physical Intelligence

**Published:** 1805-08-01

**Authors:** 


### MEDICAL AND PHYSICAL INTELLIGENCE.

[ FOREIGN AND DOMESTIC. ]

*The following interesting Paper having been received too late to be inserted in the body of the Journal, the Editors present it to their Readers as the leading article of the Medical Intelligence.*

To Dr. BRADLEY.

Dear Sir,

If the following History, which I received by the last packet, from a correspondent who has been a medical practitioner in Jamaica for upwards of thirty years, should be thought of sufficient importance to merit a place in the Medical and Physical Journal, it is at your service. I am, &c.

*Clerkenwell, July* 24, 1805. W. CHAMBERLAINE.

### Case of Yellow Fever, cured by sweating in the Steam of hot Sugar.

On Wednesday morning last I was called to a young lad about fifteen years old, arrived about three months from London. I found on him every symptom of yellow fever; pulse 110; dry, husky skin, great pain in the head, thirst, &c. I immediately gave him ten grains of calomel and one drachm of jalap, which, after waiting four hours, only procured three motions. I then mixed three drachms of jalap in a pint of water, which gave him seven or eight motions more; his skin still dry and husky. The gentleman the lad lives with is a sugar-baker; I proposed to him to put the lad close to the steams of the coppers, which had an instantaneous and happy effect. The pulse fell from 100 to 70 in a few minutes; the sweat poured off him in streams; his head was immediately relieved; and, what is astonishing, he did not complain of being too hot, notwithstanding a breath of air could not enter the room where he was; and he was surrounded with the steam of sugar from all the coppers. At night he was put to bed; but when I went to see him early on Thursday morning, he had a little of the fever again, and his skin dry and husky. We again repeated the sweating process, and continued it until two o'clock that day (Thursday) when he put on his cloaths, came down stairs, said he was very well, and called for something to eat! and is now at his business quite hearty. All the calomel I gave him did not amount to thirty grains altogether. My reason for trying this experiment was this, and I think myself justified in the sight of God and man, if *I had not succeeded:* A number of the new comers in the the last fleet have died; and at that moment I was called, a patient of our friend ——— was lying dead, which made Mr. Gutzmer extremely unhappy for the fate of the young lad, whose father is a clergyman in London. I am now determined to adopt the sweating practice, I have seen such instant relief afforded from it in the case of that lad, and subsequently in one or two other cases. For this purpose I have procured a large vessel in my hospital, in which a person can sit down, and receive the steam of hot sugar without any inconvenience. The opening of the pores by external means has this advantage over sudorific medicines, the stomach is not at all affected. There was a Spanish physician here, who, I understand, had adopted this method and found the happy effect of it, and is, as I am informed, now on his way to America to claim the reward for the discovery of curing the yellow fever by the steams of boiling sugar. I never saw him, nor heard of him, until the recovery of this lad was spoken of about town.

*Kingston, Jamaica, May 12, 1805.*

---

Mr. Parker, in a letter to the Editors, dated Colchester, July 9, 1805, writes as follows:

"Having lately observed some remarks, in your very useful, and widely extended Journal, on the treatment of leeches, which, from their utility in the cure or alleviation of many local diseases, are now become an object of considerable importance in medical and surgical practice; on this account, and from the great demand, and frequent scarcity of this valuable curative agent, every means that can be devised for preventing its destruction becomes a desideratum: I am therefore induced to offer the following hint for insertion in your Journal. It has been recommended, and, I believe generally adopted, to apply a small portion of salt to the mouth of the leech, that it might empty itself of the blood that it had just sucked; but as this not unfrequently occasions its death, or at best renders it weakly some time, I have lately been in the habit of taking hold of the tail, with the finger and thumb of my left hand, and gently drawing it through those of the other, so as to evacuate the blood; and by these means it is not punished by the nauseating effect of the salt, nor is it much injured by the operation, and becomes by next day fit for service again; which appears to me by far the most humane method of treating the animal.

"I have also to communicate a circumstance relative to the natural history of the leech, which, I think, deserves recording. On removing the cover of an old half-pint bottle, which had been untouched between four and five years, we found it half full of thick, dirty water, in which were one dead and two living leeches; the latter we put into fresh water, and in a short time they became quite lively, and were repeatedly applied to patients."

---

The

The travels undertaken by Messrs. ALEXANDER VON HUMBOLDT and AIME BONPLAND, into the interior of America, excite general interest. In fact, there are few countries so worthy of the attention and investigation of enlightened men, and few travellers have combined with the spirit of observation, and the numerous attainments and talents possessed by Mess. Von Humboldt and Bonpland, such ardour for the improvement of the sciences, such courage and success in the execution of the plan they had formed. Mess. Levrault, Scholl, and Co. have published a Prospectus of the Travels of these gentlemen, the publication of which has been committed to them by the authors. Travellers, say they, have, in general, introduced all their observations into the body of their works; M. Von Humboldt has, however, thought proper to follow a contrary method, and to treat separately of objects which are of a different nature. He is, therefore, determined first to give to the public detached collections, containing whatever relates more particularly to astronomy, geology, botany, zoology, &c. before he publishes what may properly be denominated his Travels. which will embrace every thing connected with general physics, the origin of nations, their manners, their civilization, prosperity, antiquities, commerce, and political economy. Of this portion of his observations, and the History of his Travels, he will at present publish only an abridged account, entitled Abridged Relation of Travels, between the Tropics, performed in the Interior of the new Continent, in the Years 1799, 1800, 1801, 1802, and 1803. Messrs. Humboldt and Bonpland continue publishers, being united by the ties of the most intimate friendship, having shared all the fatigues and all the dangers of this expedition, and have agreed that all their publications shall bear their names conjointly. The preface of each work will announce to which of the two each distinct part belongs. This arrangement will accelerate the enjoyment of the public, and facilitate to a greater number the means of acquiring what will demand a less advance at a time. Besides, it is not agreeable to be interrupted in the midst of a narrative, sometimes by the details of an astronomical observation, and at others by the description of a plant or an unknown animal. He will publish, at the same time, his astronomical observations, and the tables of his barometrical and geodesical measures, under the title of Collection of Astronomical Observations, and Measures executed in the New Continent; and as in his Voyage, he confines himself in mentioning an altitude to the statement of it, without saying whether it was found by the barometer, or whether it was founded on geodesical measures. M. Humboldt then collects into a separate work, all the phenomena presented by the atmosphere and the soil of the equinoctial regions. This work, the result of all the investigations undertaken by our philosopher, during his five years travels in both hemispheres, is entitled, Essay on the Geography of Plants, or physical Picture of the equinoctial Regions, founded on the Observations and and Measures taken between the Latitude of 10° South, and 10° North, in 1799, 1800, 1801, 1802, and 1803. A large plate represents a section, passing over the summit of Chimborazo, carried from the coasts of the South Sea, to the shores of Brasil. It indicates the progressive vegetation from the interior of the soil, which contains cryptogamous plants, to the perpetual snows which are the limits of all vegetation. Among these is distinguished the vegetation of palm-trees, &c. that of fern-trees, quinquina, and gramineous plants. The name of each plant is written at the height at which it is found, according to the measures determined by M. Von Humboldt. Fourteen scales, placed on each side of the table, relate to the chemical composition of the air, of its temperature, of its hygrascopical and cyanometrical state, of the electrical phenomena, of the horizontal refraction, of the decrease of gravitation, of the culture of the soil, of the height at which the different kinds of tropical animals live, &c. It is, without doubt, the most general physical table, of any portion of the globe ever attempted. The same booksellers are likewise printing two other works, which belong to descriptive natural history; one on botany and the other on zoology. The herbary which these travellers brought from Mexico, the Cordilleras of the Andes, the Oronoko, Rio Negro, and the river of Amazons, is one of the richest in exotic plants that was ever conveyed to Europe. Having long resided in countries which no botanist had ever visited before them, it is easy to conceive how many new genera and species there must be among the 6300 kinds, which they collected under the tropics of the new continent. Were they to publish at once the systematic description of all these vegetables, they would employ several years in ascertaining what is really new, or they would run the risk of publishing, under new names, plants already known. It therefore appeared preferable to give, without any regular order, the designs of the new genera and species, which they have been able sufficiently to determine, and to publish at a subsequent period, a work without plates, which contain the diagnoses of all the species, systematically arranged. It is with this view that they publish the Equinoctial Plants collected in Mexico, the island of Cuba, the Provinces of Caraccas, Cumana, and Barcelona, in the Andes of New Grenada, Quito and Peru, on the Banks of Rio Negro, the Oronoko, and the River of Amazons.

Messrs. Humboldt and Bonpland have been equally fortunate in making discoveries in zoology and comparative anatomy. They have collected, in great numbers, descriptions of animals, hitherto unknown; monkies, birds, fish, amphibious animals; for example, the axaloti of the lakes of Mexico, a problematical animal of a nature similar to the cameleon. M. Von Humboldt has made drawings of numerous objects of comparative anatomy, relative to the crocodile, the sea-cow, the sloth, the lama, and the larynx of monkies and birds. He has brought over a collection of sculls of Indians, Mexicans, Peruvians, and natives of the banks of the

Oronoko;

Oronoko; and these drawings are not less interesting for the history of the different races of our species than for anatomy. These materials, among which will be found a notice on the fossile elephants' teeth found at the elevation of 2600 yards above the sea, will appear in numbers, under the title of Collection of Observations in Zoology and comparative Anatomy, made during Travels between the Tropics While these various works are in the course of publication, M. Von Humboldt will complete the engraving of the Geological Atlas of the Cordilleras of the Andes and of Mexico, containing profiles founded on measured heights; of the Essay on geological Pasigraphy, or on the manner of representing the phenomena of the stratification of the rocks, by perfectly simple signs; and of the Geographical Atlas, which will contain a map of the river la Madelaine, in four plates; others of the Oronoko, Rio Negro and Cassiquiare, and the general map of the kingdom of New Spain: the latter will be accompanied with a statistical account of the country. All these maps were drawn by M. Von Humboldt himself, from his own astronomical observations, and a great number of interesting materials which he collected. He will, at the same time, put the finishing hand to the first volume of his Travels. To the subjects already mentioned, as being particularly treated of in that work, should be added, observations on the climate relative to organisation in general; considerations on the ancient state of civilization of these regions, and detailed notices on the management and produce of the mines. A folio volume of engravings will exhibit several views of the Cordilleras, and valuable designs of the antiquities of Mexico and Peru, such as the elegant arabesques which cover the ruins of the ancient palace, several enormous pyramids constructed of brick, statues, and chronological monuments, which have a very striking analogy to those antiquities of Indostan with which we are acquainted. Several of these plates are already engraved with great care. As M. Von Humboldt publishes these different works at the same time in German and French, both editions may be considered as originals. An English translation is preparing under the inspection of the author. The equinoctial Plants, by M. Bonpland, will appear only in French; a great part of the text being in Latin, it will therefore be understood by the literati of all Europe.

The following is a list of their works, which are either in the course of publication or shortly will issue from the press:— Abridged Narrative of Travels between the Tropics, performed in the Interior of the New Continent during the Years 1799, 1800, 1801, 1802, and 1803, quarto, which was to appear in the month of July. Collection of astronomical observations, and measures executed in the New Continent, same size and paper, to appear in the course of the present year. Essay on the Geography of Plants; or Physical Picture of the equinoctial Regions, founded on Observations and Measures taken between the Latitude of 18° South, and 10° North, in 1799, 1800, 1801, 1802, and 1803, quarto, with one plate. Equinoctial Plants collected in Mexico, the Island of Cuba, the Provinces of Caraccas, Cumana, and Barcelona, in the

Andes of New Granada, Quito, and Peru, on the Banks of the Rio Negro, Oronoko, and the River of the Amazons, with plates, folio. Collection of Observations in Zoology and Comparative Anatomy, made during Travels between the Tropics, quarto, with plates. All these works collectively will bear the general title of Travels of Messrs. Alexander Von Rumboldt and Aime Bonpland. They will all be printed uniformly, excepting the Equinoctial Plants, for which a larger size was required on account of the figures.

---

*Permanent and portable Apparatus for purifying the Air.*

An apparatus for purifying the air, on the principles of Guyton Morveau, has been lately introduced into several of the hospitals at Paris, and the departments, which possesses the advantage of being portable, and of retaining its properties for a considerable time. It consists of a vessel of thick glass, containing about six decilitres ( 1¼ wine pint nearly.) The edge of the vessel, which is very strong, is ground very accurately, and covered with a disk of glass, which seals it hermetically.

This vessel is fixed in a small plank, which supports a frame of wood, in the form of a press, and is provided with a screw to raise or lower the plate of glass, in order to open or shut the apparatus at pleasure.

To produce the disinfecting gas, 40 grammes ( 1¼ oz. ) of black oxide of manganese, powdered and passed through a hair sieve, is put into the vessel; afterwards one decilitre (⅙ of the capacity) of pure nitric acid, of 1. 40. specific gravity, and an equal volume of muriatic acid of 1. 13. specific gravity, is poured over it.

When the mixture is made, the glass cover is pressed strongly down by means of the screw, care being taken that there is no dirt on the edge of the vessel to prevent it from fitting close. Two-thirds of the vessel must always be kept empty to contain the gas.

To purify any place whatever, it is sufficient to unscrew one turn of the pressure screw, and to leave the apparatus open one or two minutes, according to the size of the place to be purified: the expansion of the gas will be soon perceived through the whole of the apartment: the apparatus is then to be closed.

The effects of this apparatus will continue for about six months, using it daily: and when they cease, the vessel is emptied and washed out, and the ingredients renewed in the proportions indicated.

It is of great utility in purifying the air of hospitals, prisons, workshops, &c. where the number of persons, or any other cause, renders such a measure necessary. The only precaution the use of it requires is, to avoid the spontaneous respiration of the gas immediately on its issuing from the vessel, which, without being dangerous, would be disagreeable.

Similar apparatuses on a smaller scale are also made, which are enclosed in a box-wood case, and carried in the pocket.

---

A member

A member of the society of sciences and belles-lettres at Douy, has lately published a simple process for purifying water. He takes a common garden pot, in the middle of which he places a piece of wicker work; on this he spreads a layer of charcoal of four or five inches in thickness, and above the charcoal a quantity of sand. The surface of the sand is covered with paper pierced full of holes, to prevent the water from making channels in it. This filter is to be renewed occasionally. By this process, which is at once simple and economical, every person is enabled to procure pure limpid water at a very trifling expence.

---

A sulphur spring, of extraordinary strength and medical powers, was lately discovered near Darlington, in the county of Durham, upon Mr. Lambton's estate, by some men employed by him to bore in search of coal. Baths have been erected upon the spot, which, from the character the water has already acquired, are resorted to with great eagerness.—We understand that Mr. Peacock, of Darlington, is about to publish an analysis of this water, with its history and medical effects.

---

A Committee of the Medical Council of the Royal Jennerian Society, has been appointed to enquire into the nature and evidence of those cases of small-pox, which are said to have taken place subsequently to cow-pox, and have excited prejudices against vaccine inoculation.

---

A new Society has been lately instituted, under the title of the Medical and Chirurgical Society of London; the leading objects of which are to promote a spirit of harmony among the members of the profession, and to serve as a centre for the communication of papers, which from time to time will be given to the public. The following names of the officers and council will justify the highest expectations of the advantages to science which are likely to result from this institution: President, William Saunders, M. D. F. R. S; John Abernethy, Esq. F. R. S. Vice-president; Charles Rochemont Aikin, Esq. Secretary; William Babbington, M. D. F. R. S. Vice-President; Matthew Bailie, M. D. F. R. S, Thomas Bateman, M. D. F. L. S. Gilbert Blane, M. D. F. R. S. Sir William Blizard, F. R. S. Vice-President; John Cooke, M. D. F. A. S. Vice-President; Astley Cooper, Esq. F. R. S. Treasurer; James Curry, M. D. F. A. S. Sir Walter Farquhar, Bart. M. D. Thompson Forster, Esq. Algernon Frampton, M. D. John Heaviside, Esq. F. R. S. Alexander Marcet, M. D. For. Sec. David Pitcairne, M. D. F. R. S. Henry Revell Reynolds, M. D. F. R. S. H. Leigh Thomas, Esq. James Wilson, Esq. F. R. S. John Yelloly, M. D. Secretary.

---

A Board of Health has lately been established for the purpose of preparing and digesting regulations for the most speedy and effectual modes of guarding against the introduction and spreading of of infection, and for the purifying any ship or house in case any contagious disorder should manifest itself in any part of the United Kingdom. This Board is to hold its meetings at Somerset Place, and it is composed of Sir Andrew Snape Hammond, Sir Lucas Pepys, Dr. Reynolds, Sir Francis Milman, Dr. Hunter, Dr. Heberden, Sir Alexander Munro, and Dr. Harness.

---

A catalogue of the Medical and Physical Library of the late Prof. BALDINGER, of Marburg, has been published. He was, perhaps, the most curious man in Germany with respect to every thing connected wlth the medical science. His library comprehends 16,000 volumes, exclusive of detached dissertations, treatises, or memoirs. The number of editions he possessed of the Aphorisms of Hippocrates alone exceeded one hundred; but the most remarkable circumstance connected with his library is, that it is not destitute of any necessary or essential work. The proprietor was fifty years in collecting it, and his heirs wish to dispose of it, if possible, entire.

---

Dr. Kinglake intends publishing, with all convenient speed, an extensive variety of additional cases, in farther proof of the *salutary efficacy* of the refrigerant treatment of Gout, with illustrative annotations, remarks on the present state and future prospects of the practice; general observations on legitimate criticism, and particular comments on the licentious strictures of some Reviewers on the subject.

Dr. K. requests those of his correspondents, and others who may possess any practical intelligence on the refrigerant treatment of Gout, worthy of public attention, and which they would wish should appear in this work, to transmit it to him without delay, that it might find due admission.

---

Dr. ARNEMAN, of Hamburgh, late Professor of Medicine in the University of Gottingen, and member of most of the Philosophical and Medical Societies in Europe and America, has undertaken to superintend the foreign department of the MEDICAL and PHYSICAL JOURNAL, vacant by the decease of the late Dr. NOEHDEN.

